# Immortality of the Eye

**Published:** 1875-03

**Authors:** 


### IMMORTALITY OF THE EYE.

Of course, we do not, in this connection, use that great word in its full, unmeasured, and incomprehensible significance. As such, it is inapplicable to material objects; and, least of all, to the animal organism, which is constantly undergoing change. But we speak of the eye as the feature, which, more than any other, retains through life its peculiar characteristics. It is nearer immortal than any other part of the face.

By it we often recognize friends from whom we have been separated many years, and who have changed so much, in all but the eye, that we can detect no likeness to their former selves.

Not long ago, we met an old friend whom we had not seen for twenty-five years. We had been boys together, had attended the same village school, and were in the same class at the Medical College. Our friend is now one of the leading physicians in the city of Washington. Learning that he was in this city, we called to see him. Thinking of our old schoolmate and classmate—a lively, jolly, mischievous fellow he was—we felt a chill of disappointment as we stood in the presence of a portly, dignified gentleman, and pronounced our name. He greeted us kindly, but it was not the face or the form that we expected to see; it was not the voice that we had come to hear; there was nothing in the whole appearance of the man that was like the student whom we remembered. Nothing? Not until he laughed; and then came that same old, genial, merry twinkle of the eye, which, in the schoolhouse and at the village gathering, always promised fun. *That* was enough to establish the identity of our old friend.

We often meet in the streets young men whom we knew ten or fifteen years ago as boys. They have changed from childhood to manhood. The boyish face is now covered with the manly beard, cut perhaps into some of the many fantastic and inconsistent styles of whiskers. We say the boy has grown entirely out of our recollection; we could not recognize a single lineament of his countenance; but when we detect that "something in the eye," which we can neither describe nor forget, then we know him.

Whether we take the period of life reaching from childhood to manhood, or the twenty-five or thirty years of maturity, before the individual lapses into the decadence of age, we find that, in the changes wrought by all these years, the eye is affected last and least. In it the sparkle of childhood mingles with the fire of youth, and both give peculiar lustre to the power and "expression" of the eye of the man.

It is said that "the eyes are the windows of the soul." Through them the soul looks out from its earthly house; through them it speaks without the aid of words. Perhaps because they are so intimately connected with the soul, and seem to be its favorite organs, they take from that ever-living spirit a little tinge of immortality.

---

The upper current of society presents no certain criterion by which we can judge of the direction in which the under current flows.

